# Distinct Neural Dynamics of Spatial Transformations: Egocentric Perspective-Taking and Allocentric Rotation

**DOI:** 10.3390/brainsci16060605

**Published:** 2026-06-01

**Authors:** Ido Amihai, Michael Kozhevnikov, Maria Kozhevnikov

**Affiliations:** 1 ABB AG, 68309 Mannheim, Germany; ido.amihai@de.abb.com; 2 Department of Engineering, Norfolk State University, Norfolk, VA 23504, USA; mkozhevnikov@nsu.edu; 3 Psychology Department, National University of Singapore, Singapore 117572, Singapore; 4 Martinos Center for Biomedical Imaging, Harvard Medical School, Charlestown, MA 02129, USA

**Keywords:** spatial transformations, egocentric perspective-taking, allocentric mental rotation, visual–spatial working memory, event-related potentials (ERPs), self-motion simulation

## Abstract

**Highlights:**

**What are the main findings?**

- Both egocentric and allocentric transformations show rotation-dependent ERP modulation, indicating angle-sensitive transformation processes in both tasks.
- Allocentric array rotation elicits parieto–occipital ERP effects (≈460–510 ms), whereas egocentric perspective-taking shows left-central effects (≈400–470 ms, 520–610 ms) and strong front–back asymmetry.

**What are the implications of the main findings?**

- Egocentric perspective-taking is not implemented through the posterior visuospatial rotation mechanisms characteristic of allocentric transformations.
- Instead, it reflects updating of an observer-centered reference frame, consistent with simulated self-motion processes involving vestibular and proprioceptive systems.

**Abstract:**

**Background/Objectives:** Egocentric and allocentric spatial transformations are central to spatial cognition, yet it is unknown whether they rely on the same neural mechanisms. The goal of this study was to examine whether egocentric transformations engage the neural processes associated with mental rotation in visual–spatial working memory. **Methods:** High-density EEG was recorded while participants performed two matched pointing-direction tasks, in which they indicated the direction toward a target location, while instructed to use either allocentric array rotation or egocentric perspective-taking. Response times and accuracy were recorded, and event-related potential (ERP) responses were analyzed as a function of rotation angle (100° vs. 160°) and differences between front and back pointing directions. **Results:** Response times increased with rotation angle in both tasks, whereas a front–back asymmetry in accuracy was observed only in perspective-taking. Both tasks showed rotation-related ERP modulation, but the timing and spatial distribution of these effects differed across tasks. In the array-rotation task, rotation-related ERP effects were observed over right-parieto–occipital regions at 460–510 ms. In the perspective-taking task, the ERP effects were observed over left-central regions at 400–470 ms and 520–610 ms. ERP differences between front and back directions were robust and widespread in the egocentric condition but limited in the allocentric condition. **Conclusions:** Perspective-taking does not show the posterior rotation-related ERP effect associated with mental rotation of object representations in visual–spatial working memory. Instead, it appears to reflect updating of the observer-centered reference frame, consistent with simulated self-motion processes involving vestibular and proprioceptive signals.

## 1. Introduction

The ability to represent and mentally transform the spatial locations of objects is essential for predicting object positions, interacting with moving objects, and planning goal-directed actions. Contemporary models of spatial cognition distinguish between two primary reference frames for representing object locations: egocentric representations, which encode objects’ positions relative to the observer’s body or viewpoint, and allocentric representations, which encode spatial relations among objects and environmental structures independent of the observer [1,2,3,4]. Egocentric representations are critical in contexts where performance requires continuous updating of spatial information relative to the self, including embodied tasks such as dentistry, teleoperation, and real-world activities, such as driving, dancing, and collaborative spatial tasks [5,6,7,8]. In contrast, allocentric representations encode spatial structure independently of the observer and play an important role in domains such as engineering, architectural design, and map-based navigation, where performance depends on stable, observer-independent representations of spatial layouts [9,10,11]. Both egocentric and allocentric representations play a significant role in navigation, but serve different functions: allocentric coding supports stable mapping of spatial layouts, whereas egocentric coding enables continuous transformation of heading, position, and action-relevant vectors during movement, including path integration and goal-directed reorientation [3,4,7,12,13].

Efficient spatial behavior requires not only maintaining egocentric and allocentric representations but also transforming spatial information within each reference frame as viewpoints or object configurations change. Behavioral studies investigating spatial transformations have often employed pointing-direction tasks that require participants to compute object locations using either egocentric or allocentric transformations [7,14,15]. In egocentric pointing tasks, typically referred to as perspective-taking tasks, participants imagine adopting a different viewpoint within a spatial layout and then indicate the direction of a target object relative to that imagined perspective. In contrast, allocentric pointing tasks, often referred to as array-rotation tasks, require participants to mentally rotate the spatial configuration of objects while maintaining their own viewpoint fixed. Although both tasks involve spatial transformations, they differ in the reference frame that must be manipulated during the computation of object locations. In particular, performance differs systematically between these two types of spatial transformation. In egocentric perspective-taking tasks, pointing accuracy is typically higher for targets in front of the imagined viewpoint than for those behind it, producing a consistent front–back asymmetry in pointing errors. In contrast, allocentric array-rotation tasks generally do not exhibit this asymmetry [7,14,15]. These behavioral differences suggest that egocentric and allocentric spatial transformations rely on partially distinct cognitive processes.

Neurobiological studies examining egocentric and allocentric spatial transformations, however, have produced mixed findings regarding the neural correlates of egocentric versus allocentric transformation. Early neuropsychological findings revealed that people with lesions in the right posterior cortex demonstrated impairments in allocentric transformations, whereas selective impairment in egocentric transformations was observed after damage to the left posterior cortex [16,17]. Similarly, subsequent neuroimaging studies suggested that distinct neural systems are engaged for different types of spatial transformation. For example, Committeri et al. [18] reported largely distinct cortical systems associated with the two reference frames, such that egocentric spatial judgments preferentially engaged a dorsal parietal network (superior parietal lobule and intraparietal sulcus, extending into the precuneus), whereas allocentric judgments recruited a medial temporal–retrosplenial system, including the parahippocampal and retrosplenial cortex. Similarly, Zacks et al. [19] compared imagined self-rotation with object-based mental rotation and reported different dominant regions for the two transformations: object-based transformations led to selective increases in right parietal cortex and decreases in left parietal cortex, whereas perspective transformations led to selective increases in left temporal cortex. Wraga et al. [20] reported that imagined self-rotation and object rotation engage partially distinct neural systems, with object rotation leading to a spread of the BOLD signal from left premotor areas to the left primary motor cortex, while self-rotation activated the left supplementary motor area. Other studies, however, reported greater overlap between the neural systems supporting these transformations. Zaehle et al. [21] showed that egocentric spatial processing requires a subsystem of the allocentric processing resources: Egocentric spatial relations are mediated by medial superior-posterior areas, whereas allocentric spatial coding additionally involves right parietal cortex, the ventral visual stream, and the hippocampal formation. Lambrey et al. [22] reported that both viewpoint and array rotations engaged a common network including bilateral superior and inferior parietal cortex and the precuneus, with relatively small differences between the tasks, concluding that spatial transformations involve translation between egocentric and allocentric reference frames rather than relying on separate neural mechanisms. The authors interpret these findings as supporting a model in which the parieto-occipital sulcus/retrosplenial cortex mediates spatial updating through transformations between egocentric and allocentric reference frames. Computational models of spatial cognition propose that egocentric parietal and allocentric medial temporal representations are dynamically linked via transformations mediated by parietal and retrosplenial systems rather than implemented as independent processes [23]. Taken together, neuroimaging findings provide no clear consensus regarding whether egocentric perspective transformations and allocentric array rotations rely on distinct neural mechanisms or are implemented within largely overlapping spatial transformation networks.

Complementing neuroimaging approaches, several event-related potential (ERP) studies have examined the temporal dynamics of spatial transformations during mental rotation tasks. These studies have consistently shown that increasing rotation angles is associated with reductions in ERP mean amplitude between approximately 400 and 800 ms after stimulus onset, most prominently over parieto-occipital electrode sites [24,25,26,27]. This ERP modulation has been interpreted as a neural correlate of the imagined rotation processes involved in allocentric spatial transformations [28]. However, previous ERP studies have primarily focused on object-based mental rotation and allocentric transformations, leaving the neural dynamics of egocentric perspective transformations largely unexplored.

One way to understand whether these spatial transformations rely on similar or distinct neural mechanisms is to examine their temporal dynamics. While neuroimaging studies provide important information about the spatial localization of neural activity, they offer limited insight into the temporal sequence of processes involved in spatial transformations. ERPs, with their high temporal resolution, provide a complementary approach for examining the neural dynamics underlying egocentric and allocentric transformations. In contrast to object-based mental rotation, which can be performed as a holistic transformation of the array, egocentric perspective-taking involves at least two component processes: first, reorienting oneself to a new imagined heading, and second, determining the direction of a target object from that updated viewpoint. The first goal of the present study was therefore to test whether the self-reorientation component of perspective-taking shows rotation-related ERP modulation as a function of angular disparity. Behavioral studies demonstrate that response times in perspective-taking tasks increase systematically with rotation angle [7,29,30], similar to that observed in classic object-rotation tasks, indicating that the self-reorientation process depends on the magnitude of the angular transformation. Based on this, we predicted that increasing the self-rotation angle would modulate ERP activity during perspective-taking in a manner comparable to that observed in allocentric spatial transformations. The second goal of this study was to examine neural processes associated with the directional judgment component of perspective-taking. Prior behavioral work has demonstrated systematic differences in error patterns between egocentric and allocentric pointing-direction tasks, particularly the front–back asymmetry observed in egocentric perspective-taking [14,31]. To determine whether these differences are reflected in neural dynamics, we compared ERP responses associated with front and back pointing directions across tasks.

## 2. Materials and Methods

### 2.1. Participants

Twenty participants (12 female, mean age = 23.5 years, range: 21–32 years) were recruited for the study. The study was approved by the National University of Singapore Department of Psychology Ethics Review Committee, and all participants provided informed consent prior to the experiment.

### 2.2. Materials

Each participant completed two computerized versions of the Pointing Direction Task (Array-Rotation and Perspective-Taking), designed to tap allocentric and egocentric spatial transformations, respectively. These two versions were revised versions of those used in ref. [7], which were in turn adapted from the paper-and-pencil versions developed by Kozhevnikov and Hegarty [15]. Each task version consisted of 72 trials.

**Figure 1:**
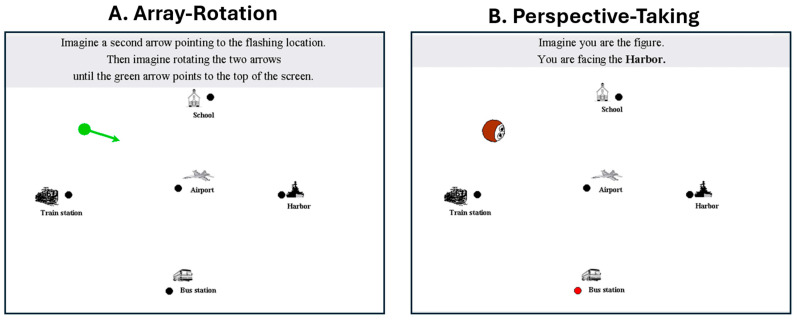
Pointing direction task. Subjects were presented with an array of objects with an adjacent black dot. In the Array-Rotation task (Panel (**A**)), there was also a green arrow, and in the Perspective-Taking task (Panel (**B**)), a bird’s-eye view of a face. During the Array-Rotation task (Panel (**A**)), all dots were black; during Stage 2 of the Perspective-Taking task (Panel (**B**)), one of the dots became red and began blinking. The task was to indicate the location of the dot relative to the direction that the face is gazing or the direction of the arrow.

Each trial consisted of two stages. In the first stage (Stage 1), participants saw a picture of a layout of images that represent different locations (see Figure 1A). The layout consisted of a starting location (for Array-Rotation, a green arrow with a circle base, and for Perspective-Taking, a picture of a character’s head) and five other locations (e.g., airport, school, etc.). All five locations were represented as black points, with each point labeled with the location name and a small pictogram. For each trial, the instructions were displayed at the top of the screen, and an array of response keys, with which participants were to indicate their responses, was displayed at the bottom of the screen. At this stage, for the Array-Rotation version of the task, participants simply had to look at the layout, while for the Perspective-Taking version, participants were to imagine taking the perspective of the character on the screen.

The start of the second stage (Stage 2, see Figure 1B for perspective-taking) was marked by a change in color from black to red and the subsequent flashing of one of the five location points five seconds later. At this stage, for Array-Rotation, participants were to imagine a second arrow emerging from the base of the green arrow and pointing to the flashing location. Then participants were to imagine rotating the angle composed from these two arrows until the first arrow pointed vertically up (i.e., was aligned with the vertical axis of the computer screen). After the array rotation, participants were to indicate the pointing direction of the second arrow by clicking the corresponding response key. For Perspective-Taking, participants were to imagine pointing to the flashing location from their newly imagined perspective (the character on the screen) and click the response key corresponding to the pointing direction.

The imagined rotations varied from 100° to 260° (relative to the upright direction) in increments of 20°. Angles less than 100° and more than 260° were not used for imagined headings because previous research has shown that observers usually used strategies other than egocentric strategies for those angles (e.g., analytical strategies or tilting the head to ‘see’ the angle) [15]. For all analyses, the same rotation angles in clockwise and counterclockwise directions (e.g., 100° and 260°) were averaged together as the same condition (100°). This approach is based on evidence that participants perform egocentric transformations along the shortest rotational path [15]. Furthermore, for each of the 9 imagined rotations, all 8 of the cardinal and inter-cardinal pointing directions (0°/Front, 45°/Front-Right, 90°/Right, 135°/Back-Right, 180°/Back, 225°/Back-Left, 270°/Left, 315°/Front-Left) were used, resulting in 72 trials in each block of the task.

### 2.3. Procedure

All participants performed both versions of the Pointing Direction Task (Array-Rotation and Perspective-Taking). Participants always performed 2 consecutive blocks of each version, with the order counterbalanced across subjects. At the beginning of each block, the task instructions were explained to the participants and displayed on the computer screen. Moreover, the participants performed 6 practice trials, which were not analyzed, prior to the first Array-Rotation and Perspective-Taking blocks. EEG was recorded throughout the experiment.

### 2.4. EEG Data Acquisition and Analysis

EEG was recorded using a 256-channel HydroCel Geodesic Sensor Net (Electrical Geodesics, Inc., Eugene, OR, USA), and all electrodes were referenced to CZ during recording. Signals were continuously sampled at 250 Hz, amplified using the EGI NetAmps 300 amplifier (Electrical Geodesics, Inc.), and stored for offline analysis.

EEG data were processed using EEGLAB version 2019.1, a MATLAB toolbox [32]. The EEG data were re-referenced to the average reference and band-pass filtered at 0.1–30 Hz. The signal was then cleaned of eye-blink artifacts using Independent Component Analysis [33]. Segments contaminated by other artifacts were detected as amplitudes exceeding ±100 µV or activity below 0.5 µV that spanned over 100 ms (which was never observed) in any channel. Additional artifacts were removed through manual inspection.

The EEG was then segmented into 1100 ms long epochs starting 100 ms prior to the onset of Stage 1 and the onset of Stage 2. Each segment was averaged separately, and baseline correction was adjusted by subtracting the mean amplitude of the pre-stimulus period of each ERP from all the data points in the segment. Because the temporal and spatial distribution of potential ERP differences was not known a priori, a cluster-based non-parametric permutation approach was employed [34], which allows statistical evaluation across electrodes and time points while controlling for multiple comparisons. For this analysis, electrodes were first grouped into nine scalp regions of interest based on anterior–posterior position and laterality, and permutation testing was then applied across scalp regions and time points.

### 2.5. Analysis of Effects of Required Angular Transformation

The EEG for 100° and 160° angle rotations was segmented separately for Array-Rotation and Perspective-Taking trials. The analysis was restricted to 100° and 160° to compare neural responses between conditions at angles that produce robust behavioral differentiation. These large-angle conditions were selected based on prior spatial transformation research showing that larger angular disparities reliably engage perspective-taking processes and produce substantial behavioral differentiation [7,15]. Given that performance varies gradually with angular disparity, a parametric analysis across all angles would address the effect of angle rather than the contrast between tasks. The selected angles, therefore, provide a controlled basis for comparing Perspective-Taking and Array-Rotation without additional variability from intermediate angles.

The segments were time-locked to the onset of the rotation task: for Array-Rotation, they were time-locked to the onset of Stage 2; for Perspective-Taking, they were time-locked to the onset of Stage 1. The temporal alignment was defined relative to the onset of the spatial transformation in each task, which occurs at Stage 1 in Perspective-Taking (adoption of the imagined viewpoint) and at Stage 2 in Array-Rotation (presentation of the rotated array).

To reduce the number of statistical comparisons and control for multiple comparisons, EEG electrodes were grouped into nine regions of interest based on scalp location, defined by the combination of anterior–posterior position (frontal, central, parieto-occipital) and laterality (left, midline, right), following Mudrik et al. [35]. Subsequently, to investigate the occurrence of imagined rotations in egocentric and allocentric transformations, we computed the mean activity during the 400–800 latency region, which was assessed in a 2 (Task: Array-Rotation/Perspective-Taking) × 9 (Location: Frontal-Left/Frontal-Center/Frontal-Right/Central-Left/Central-Center/Central-Right/Parieto-Occipital-Left/Parieto-Occipital-Center/Parieto-Occipital-Right) × 2 (Rotation: 100°/160°) repeated measures ANOVA. To investigate additional differences between allocentric and egocentric rotations, we used the cluster-based nonparametric permutation test described in ref. [34], separately for the Array-Rotation and Perspective-Taking tasks using all 9 regions (for a complete explanation of the statistical procedures, see refs. [34,35]. Since previous studies on array rotations found that differences in the degree of rotation lead to differences in the mean ERP amplitudes at 400–800 ms latencies so that larger rotations lead to more negative amplitudes (e.g., [24,25]; see ref. [28] for a review), we first compared the ERPs elicited by 100° and 160° rotations during this time period for both tasks. Subsequently, to explore additional differences between egocentric and allocentric rotations, we performed the same analysis using data points from the entire 1000 ms trial.

### 2.6. Analysis of Effects of Pointing Direction

The EEG for front and back pointing directions was segmented separately for Array-Rotation and Perspective-Taking and time-locked to the onset of Stage 2. The same cluster-based nonparametric statistical permutation test used for the rotation analyses was applied to compare differences between front and back directions, separately for Array-Rotation and Perspective-Taking, across all 9 regions and the entire 1000 ms trials.

## 3. Results

### 3.1. Data Selection

The two task versions of the Pointing Direction Task are intentionally designed to be as similar as possible in terms of stimuli, angular transformations, and number of trials, differing only in the instructions that require either object-based rotation (Array-Rotation) or imagined changes in self-orientation (Perspective-Taking). This design enables a direct comparison of allocentric and egocentric transformations while controlling perceptual and task demands. However, because the stimulus structure is identical across conditions, participants may, in principle, apply the same strategy to both tasks. In particular, it is well established that perspective-taking tasks are often solved using allocentric strategies, for example, by mentally rotating the stimulus configuration rather than transforming one’s own viewpoint [36,37]. To ensure that participants follow the intended transformation strategy in each condition, we therefore apply an objective behavioral criterion based on established differences between egocentric and allocentric processing. Previous work has shown that when imagining taking a particular perspective in a real-world environment, people are faster and more accurate in naming and pointing to objects in front of them than to those behind them, whereas this asymmetry is not expected for allocentric array-rotation performance [7,14,31]. The front–back asymmetry thus provides a critical behavioral index of whether the intended egocentric strategy is used. To minimize this potential confound and reduce between-participant variability in strategy use, we include only participants whose front–back accuracy difference is greater in the Perspective-Taking condition than in the Array-Rotation condition (n = 13). This selection reduces between-participant variability in the cognitive process of interest and increases the sensitivity of the within-subject comparison. In addition, EEG data from three participants were excluded due to technical recording issues, resulting in a final sample of 10 participants (8 female, mean age = 22.9 years, range: 21–32 years). A sensitivity analysis for the within-subject rotation-angle contrast indicates that, with n = 10, α = 0.05, and power = 0.75, the present design can detect paired effects with approximately dz = 0.86 or larger, a magnitude consistent with the large effects reported in previous ERP studies of mental rotation. The results of the sensitivity analysis, together with the reduced variability achieved through strict behavioral control of strategy use, support the adequacy of the final sample size for the study’s primary aims.

### 3.2. Behavioral Results

Prior to behavioral analysis, trials were restricted to conditions that reliably engage the intended transformation processes. Angles less than 100° and greater than 260° were not used for imagined headings because previous research has shown that observers usually use strategies other than egocentric strategies at those angles (e.g., analytical strategies or tilting the head to ‘see’ the angle) [7,15].

**Figure 2:**
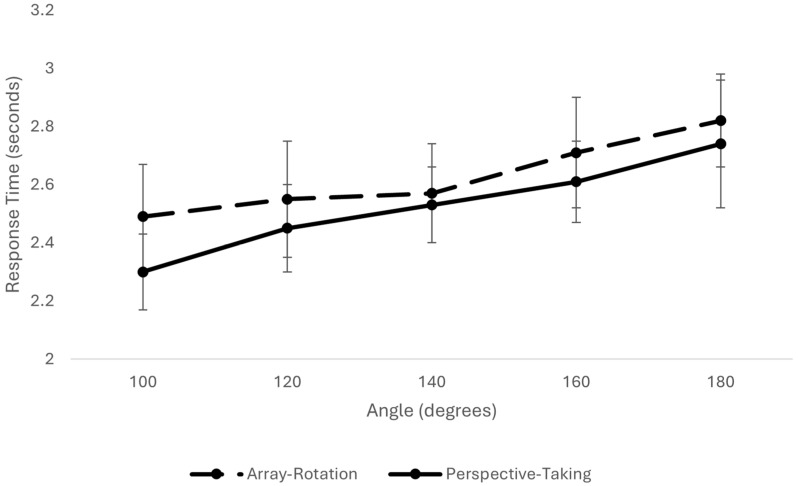
Effects of angle on response time for array-rotation and perspective-taking. Error bars represent the standard error of the mean.

A 2 (Task: Array-Rotation, Perspective-Taking) × 5 (Rotation: 100°, 120°, 140°, 160°, 180°) repeated-measures ANOVA revealed a significant main effect of Rotation, F(4, 36) = 12.38, *p* = 0.001, with response times increasing as a function of angular disparity (Figure 2). No main effect of Task or Task × Rotation interaction was observed (F < 1). Accuracy was not affected by rotation angles (all *p* > 0.40).

**Figure 3:**
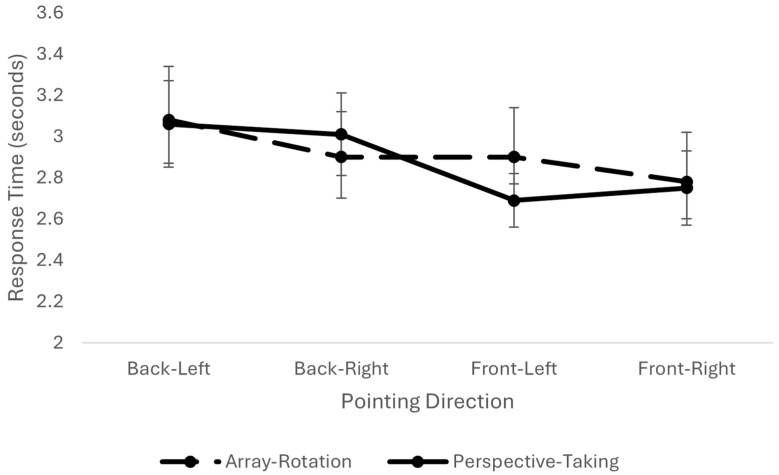
Effects of pointing direction on response time for array-rotation and perspective-taking. Error bars represent the standard error of the mean.

**Figure 4:**
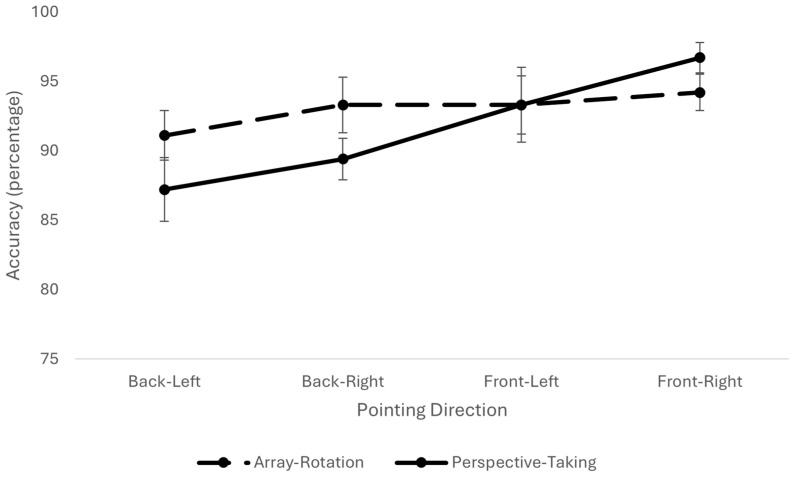
Effects of pointing direction on accuracy for array-rotation and perspective-taking. Error bars represent the standard error of the mean.

For the pointing-direction analysis, we focused on front and back directions, as these provide the critical contrast for assessing egocentric transformation processes. Indeed, as noted earlier, when imagining taking a particular perspective, people are worse at pointing to targets behind them compared to those in front [14,31]. Since front responses themselves are typically associated with high accuracy, they were not used to assess spatial error patterns within quadrants; instead, front–back differences were used to capture the characteristic asymmetry associated with perspective-taking. A 2 (Task) × 2 (Pointing Direction: Front, Back) repeated-measures ANOVA revealed a significant main effect of Pointing Direction for both response time, F(1, 9) = 14.02, *p* = 0.005, and accuracy, F(1, 9) = 13.11, *p* = 0.006, indicating differences between front and back judgments. Critically, a Task × Pointing Direction interaction was observed for accuracy, F(1, 9) = 36.13, *p* < 0.001, and approached significance for response time, F(1, 9) = 4.71, *p* = 0.058, reflecting a larger front–back difference in the Perspective-Taking condition. Follow-up analyses confirmed that the pointing-direction effect was present for Perspective-Taking, F(1, 9) = 37.30, *p* < 0.001, but not for Array-Rotation, F(1, 9) = 1.35, *p* > 0.20 (Figure 3 and Figure 4).

The behavioral results replicated the results of previous studies that demonstrated an increase in response times with increasing angles for both array-rotation and perspective-taking tasks, and a difference in accuracy between front and back pointing directions for perspective-taking tasks, but not for array-rotation tasks (e.g., [7,14]). This confirms that participants followed the instructed transformation strategy, supporting the validity of the subsequent EEG analyses.

### 3.3. ERP Results

#### 3.3.1. Effect of Angular Transformation

The 2 (Task) × 9 (Location) × 2 (Rotation) repeated-measures ANOVA on the 400–800 ms mean amplitude revealed a significant main effect of Rotation, F(1, 9) = 5.68, *p* < 0.05, with 160° transformations producing more negative amplitudes than 100° transformations. The main effect of Location was also observed, F(8, 72) = 2.39, *p* < 0.05, whereas the main effect of Task was not significant (F < 1). The Task × Location × Rotation interaction approached significance, F(8, 72) = 1.82, *p* = 0.087.Although this interaction did not reach significance, its pattern suggested that the spatial distribution of the rotation effect differed across tasks. To examine this possibility, we conducted follow-up comparisons within each task. For the Array-Rotation task, the rotation effect was significant at parieto-occipital locations (Parieto-Occipital-Central: t(9) = 2.18, *p* < 0.05; Parieto-Occipital-Right: t(9) = 2.22, *p* < 0.05, one-tailed). In contrast, for the Perspective-Taking task, the effect was observed at the Central-Left location (t(9) = 2.85, *p* < 0.05, one-tailed).

**Figure 5:**
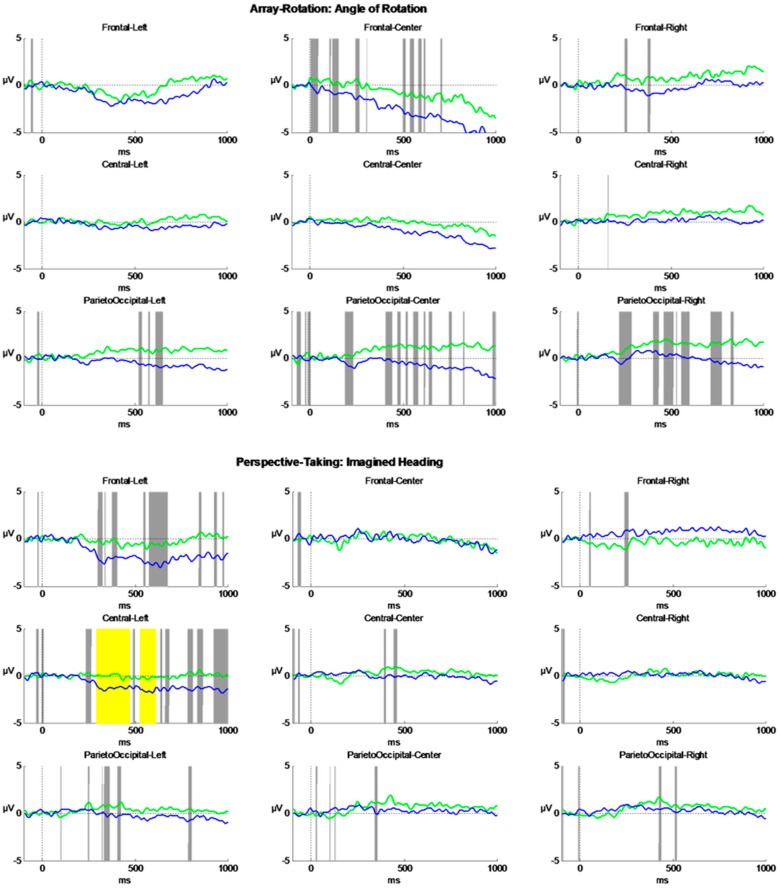
Effects of angular transformation. differences between small (100°, green lines) and large (160°, blue lines) rotation differences for the Array-Rotation (top panel) and Perspective Taking (bottom panel) tasks. Gray regions represent significant clusters at a *p* < 0.05 threshold, and yellow regions represent significant clusters after correcting for multiple comparisons.

Given that the spatial and temporal distribution of these effects was not specified a priori, and that the interaction did not provide a definitive localization, we further examined the data using cluster-based permutation analyses [34]. Consistent with the above pattern, permutation analyses revealed that rotation-related differences in the Array-Rotation task were localized to parieto-occipital regions (Figure 5, top panel), with a significant cluster in the right parieto-occipital region between 460 and 510 ms after correction for multiple comparisons. Under a less conservative threshold (*p* < 0.05), additional effects were observed predominantly within parieto-occipital areas. For the Perspective-Taking task, rotation-related differences were localized to left-central regions, with significant clusters between approximately 400–470 ms and 520–610 ms (Figure 5, bottom panel). When the entire epoch was analyzed, additional effects were observed at the left-central location between 290–470 ms and 520–610 ms. At a less conservative threshold (*p* < 0.05), these effects extended to left-frontal regions.

#### 3.3.2. Effects of Pointing Direction

**Figure 6:**
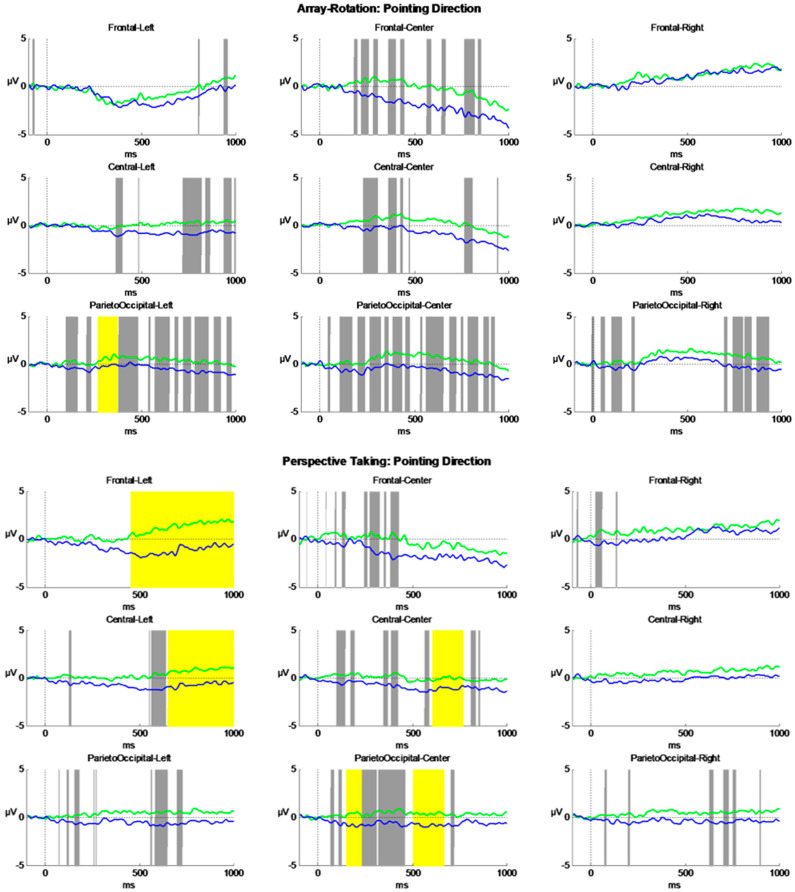
Effects of pointing direction. Differences between Back (green lines) and Front (blue lines) pointing directions for the Array-Rotation (top panel) and Perspective-Taking (bottom panel) tasks. Gray regions represent significant clusters at a *p* < 0.05 threshold, and yellow regions represent significant clusters after correcting for multiple comparisons.

The permutation test revealed larger and more extensive differences between front and back pointing directions for the Perspective-Taking task relative to the Array-Rotation task. For the Array-Rotation task, after correction for multiple comparisons, a single significant front–back difference was observed between approximately 260–370 ms at the left-parieto-occipital location. In contrast, for the Perspective-Taking task, multiple significant front–back differences were observed. These included effects at the central location (approximately 600–770 ms), left-central location (approximately 650–1000 ms), left-frontal location (approximately 450–1000 ms), and parieto-occipital-central location (approximately 150–230 ms and 500–670 ms). When a less conservative threshold (*p* < 0.05) was applied, additional significant regions were observed for both tasks across the scalp (see Figure 6).

## 4. Discussion

The present study investigated the neural dynamics underlying egocentric perspective transformations and allocentric array rotations using event-related potentials. Although both tasks required participants to compute object directions following spatial transformations, the ERP results revealed differences in their neural dynamics.

Specifically, the first goal of this study was to investigate and compare the ERP correlates of imagined mental rotations in egocentric and allocentric spatial transformations. Although the similarities in response times between Perspective-Taking and Array-Rotation tasks are suggestive of the utilization of imagined rotations during both tasks (e.g., [7,29,30]), direct evidence for this hypothesis has thus far been lacking. The present results provide such evidence by showing that both types of spatial transformations elicited significant ERP differences between 100° and 160° rotations during the 400–800 ms latency period, with larger rotations producing more negative amplitudes. This is consistent with previous findings on allocentric transformations (e.g., [24,25,27]), and indicates that egocentric transformations are also systematically modulated by rotation angle.

However, the spatial distribution of this effect differs markedly between the two tasks. Rotation-related activity in the Array-Rotation task was concentrated in parieto-occipital regions, consistent with previous ERP findings on object-based mental rotation and visual–spatial working memory processes (e.g., [24,25]). These findings are consistent with the interpretation that array rotation involves transforming the spatial representation of the object layout itself, requiring participants to mentally rotate the visual configuration of objects before computing the target direction. In contrast, Perspective-Taking did not show this posterior pattern; instead, rotation-related effects were localized to central and frontal regions, particularly over the left hemisphere, and the effect appeared to emerge earlier than in the array-rotation task. The absence of posterior ERP signatures during perspective-taking indicates that participants were not performing a rotation operation in visual–spatial working memory. Visual–spatial working memory is associated with sustained occipital–parietal activity and characteristic posterior ERP components; the lack of these signatures therefore suggests that, although both tasks involve rotation-related processing, egocentric perspective-taking is supported by a different, non-visual mechanism distinct from that underlying allocentric rotation processes. This interpretation is reflective of previous research (e.g., [38]), which showed that early visual deprivation promotes the use of egocentric spatial representations in congenitally blind individuals, whereas individuals with prior visual experience were more inclined to use allocentric spatial representations.

Egocentric perspective-taking requires the observer to update their own orientation, and the mechanism uniquely compatible with prior research for achieving this computation is the internal simulation of the self-motion signals that accompany real movements of the head and body. During actual movement, these self-motion signals track changes in orientation, with vestibular signals related to head rotation, and proprioceptive and motor-related signals encoding changes in body orientation [39,40]. Consistent with this view, neuroimaging studies show that imagining body-based transformations engages motor planning regions, including the supplementary motor and premotor cortices [41], while vestibular and body-related signals, engaging the temporo-parietal and insular cortices, contribute to the representation of body orientation in space [42]. These data are consistent with the idea that egocentric transformations rely on simulated self-motion rather than on rotating visual layouts in visual working memory (see also Ref. [43] for further review). In addition, evidence from spatial navigation research indicates that perspective-taking is closely related to path integration, the ability to track one’s position and orientation from self-motion cues. Individuals who perform better on perspective-taking tasks also perform better on blindfolded triangulation, indicating more accurate use of vestibular and proprioceptive signals, and show superior performance in wayfinding tasks that require continuous updating of orientation [12]. Within this framework, the more central and frontal ERP distribution observed during perspective-taking is consistent with engagement of systems involved in simulated self-orientation and spatial updating, rather than with posterior visual–spatial mechanisms characteristic of allocentric object rotation. Thus, although both tasks show rotation-dependent modulation, the present results indicate that while allocentric transformations operate on visual object representations, egocentric perspective-taking may rely on updating of the observer-centered reference frame, supported by neural systems associated with self-motion and body-based spatial computations.

The second goal of this study was to compare ERP responses associated with pointing-direction computations under egocentric and allocentric task demands. Consistent with prior behavioral findings, egocentric perspective-taking produced clear differences between front and back pointing directions, reflected in more negative ERP amplitudes for front directions across multiple scalp regions and time windows. Importantly, a qualitatively similar but substantially weaker effect was also observed in the allocentric Array-Rotation condition, despite the absence of corresponding behavioral differences. A likely explanation for this pattern is that participants did not rely exclusively on allocentric transformations in the Array-Rotation task, but occasionally adopted egocentric strategies, as previously documented [15]. Under this account, the residual front–back ERP differences observed in the allocentric condition reflect intermittent engagement of egocentric computations, which are not strong enough to influence overt performance but are detectable at the neural level.

Furthermore, the front–back asymmetry observed in the egocentric condition reflects a fundamental characteristic of egocentric encoding. In egocentric representations, spatial locations are defined relative to the body axes—front, back, left, and right—and positions behind the body are reliably processed less efficiently than those in front [14,31]. The broader and more pronounced ERP effects for back-pointing than front-pointing directions observed during perspective-taking are consistent with this inherent property of egocentric encoding. In contrast, allocentric transformations operate on object-to-object relations and do not depend on the observer’s orientation. Thus, they do not inherently require differential processing of front versus back directions, which explains the weaker, less clearly differentiated ERP pattern in the allocentric condition.

The distinctions between egocentric and allocentric transformations reported in this study are not only theoretically important but also have direct implications for real-world performance in tasks and professional activities—such as spatial navigation in unfamiliar environments, image-guided medical procedures, dentistry, and teleoperation—where the position and orientation of the body are critical. In such contexts, relying on egocentric, self-motion–based computations may place different demands on neural systems than allocentric, visually based transformations, and may be differentially vulnerable to fatigue, sensory degradation, or pathology. Understanding that these tasks draw on distinct computational operations, therefore, provides a framework for designing training protocols, interfaces, and assessment tools that are better aligned with the underlying mechanisms supporting body-centered spatial performance.

One limitation of the present study is the relatively small sample size, which reflects the strict inclusion criterion requiring participants to reliably exhibit the behavioral signature of egocentric processing. This constraint was essential to ensure that the perspective-taking condition was performed using the intended transformation strategy. Within this constrained sample, however, the effects were robust and consistently observed at both behavioral and ERP levels. At the same time, given the sample size, the present findings should be regarded as exploratory. Future studies with larger samples will be important for confirming the generality and reproducibility of these effects and for further characterization of the neural mechanisms underlying egocentric and allocentric spatial transformations. In addition, although the ERP measures used in the present study enabled characterization of the temporal dynamics distinguishing egocentric and allocentric transformations, they lack sufficient spatial resolution to directly evaluate the proposed involvement of embodied self-motion and motor simulation mechanisms in egocentric perspective-taking.

Despite its exploratory nature, the present study provides, to our knowledge, the first experimental dissociation of egocentric perspective-taking and allocentric transformation processes under controlled strategy conditions, revealing distinct temporal neural dynamics associated with these transformations. By combining strict criteria for isolating egocentric transformation strategies with temporally dissociable transformation stages, the present findings establish a foundation for future neuroscience research aimed at dissociating the computational and neural mechanisms underlying egocentric and allocentric transformations and examining their relationship to vestibular, proprioceptive, and motor simulation processes.

## 5. Conclusions

Despite the relatively small sample size constraint, the present findings suggest that egocentric perspective-taking and allocentric array rotation rely on distinct neural dynamics, even though both tasks involve spatial transformations that depend on rotation magnitude. The parieto-occipital activity observed during array rotation is consistent with classic allocentric visuospatial mental rotation processes operating on representations of object configurations. In contrast, in the perspective-taking task, the corresponding effect was localized to more central and frontal regions, particularly over the left scalp, indicating a different neural organization of the transformation process. This dissociation was reinforced by the pointing-direction results: a clear front–back asymmetry was present in perspective-taking, but not in array rotation, showing that only the egocentric task strongly depended on computations tied to the observer’s reference frame. Together, these findings suggest that perspective-taking involves transforming the observer’s egocentric reference frame through simulated changes in body orientation and possibly through neural systems associated with self-motion and spatial updating, rather than reflecting visual processing within visuospatial working memory.

## Data Availability

The data presented in this study are available on request from the corresponding author. The data is not publicly available due to privacy reasons.

## Abbreviations

The following abbreviations are used in this manuscript:

BOLD	Blood-Oxygen-Level-Dependent
CZ	Central “Zero-line” Electrode
EEG	Electroencephalogram
ERP	Event-Related Potential
